# The effects of local filtering processes on the structure and functioning of native plant communities in experimental urban habitats

**DOI:** 10.1002/ece3.9397

**Published:** 2022-10-17

**Authors:** Dorothy Borowy, Christopher M. Swan

**Affiliations:** ^1^ Geography and Environmental Systems Department University of Maryland Baltimore Maryland USA

**Keywords:** biodiversity, community assembly processes, functional traits, urban ecology, vacant land

## Abstract

Despite a growing literature‐base devoted to document biodiversity patterns in cities, little is known about the processes that influence these patterns, and whether they are consistent over time. In particular, numerous studies have identified the capacity of cities to host a rich diversity of plant species. This trend, however, is driven primarily by introduced species, which comprise a large proportion of the urban species pool relative to natives. Using an experimental common garden study, we assessed the relative influence of local assembly processes (i.e., soil environmental filtering and competition from spontaneous urban species) on the taxonomic and functional diversity of native plant communities sampled over four seasons in 2016–2018. Taxonomic and functional diversity exhibited different responses to local processes, supporting the general conclusion that species‐ and trait‐based measures of biodiversity offer distinct insights into community assembly dynamics. Additionally, we found that neither soil nor competition from spontaneous urban species influenced taxonomic or functional composition of native species. Functional composition, however, did shift strongly over time and was driven by community‐weighted mean differences in both measured traits (maximum height, Hmax; specific leaf area, SLA; leaf chlorophyll a fluorescence, Chl *a*) and the relative proportions of different functional groups (legumes, annual and biennial‐perennial species, C4 grasses, and forbs). By contrast, taxonomic composition only diverged between early and late seasons. Overall, our results indicate that native species are not only capable of establishing and persisting in vacant urban habitats, they can functionally respond to local filtering pressures over time. This suggests that regional dispersal limitation may be a primary factor limiting native species in urban environments. Thus, future regreening and management plans should focus on enhancing the dispersal potential of native plant species in urban environments, in order to achieve set goals for increasing native species diversity and associated ecosystem services in cities.

## Graphical abstract

We conducted experimental common garden study assessing the relative influence of local assembly processes on the taxonomic and functional diversity of native plant communities in urban environments. We found that taxonomic and functional diversity exhibited different responses to local processes over time. In addition, neither soil nor competition from spontaneous urban species influenced taxonomic or functional composition of native species, which suggests that dispersal limitation may be a primary factor limiting native species in urban environments.

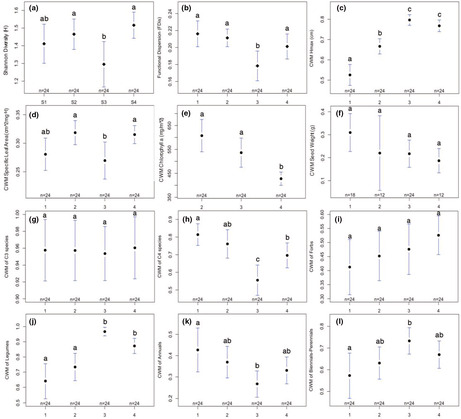

## 1 INTRODUCTION

More than 50% of the global population currently lives in cities, with an estimated increase to 68% by the year 2050 (U.N., 2018). This has led to the rapid expansion of urban landscapes, globally (Seto et al., 2012). Urbanization has profound effects on biodiversity patterns (Grimm et al., 2008) and is one of the leading causes of species extinctions (Kowarik, 1995; Marzluff, 2001; McDonald et al., 2008), with the greatest impact on biodiversity hot spots (Seto et al., 2012). Despite these changes, urban habitats often host a rich diversity of plant species (Knapp et al., 2008; Kühn et al., 2004). The composition of urban plant communities, however, is dominated by high proportions of introduced “urban exploiters” relative to native species (Aronson et al., 2014; Kowarik, 1990, 1995; Pyšek, 1998). Although this trend has been observed in many cities, leading to the characterization of urban communities as globally homogenized (i.e., exhibiting low β‐diversity across cities; McKinney & Lockwood, 1999), there still lacks a general understanding of the factors responsible for shaping these unique biodiversity patterns. Urban biotic homogenization, for example, is often attributed to competitive effects, resulting from the introduction of non‐native generalist species by humans that, over time, outcompete native residents (McKinney, 2004, 2006; but see McCune & Vellend, 2013). Other studies have hypothesized that urbanization generates unique environmental conditions and disturbance regimes, which filter out natives that do not have the functional capacity to adapt to urban environmental pressures (Williams et al., 2009). Still, other studies have concluded that the primary mechanism driving local extinctions of native plant species in urban environments is recruitment limitation caused by the proliferation of small, fragmented habitat patches in the urban landscape acting act as barriers to dispersal (Schleicher et al., 2011).

Understanding the nature of community assembly processes underlying contemporary biodiversity patterns in cities is necessary for reconciling these different conclusions. More importantly, it is vital for the development of general principles that can predict how communities will respond to urban pressures over time (Swan et al., 2011), which directly influence management and regreening efforts (Niemelä, 1999). Urban regreening approaches often consist of physically introducing focal species via seed additions, altering environmental conditions (e.g., soil amendments), or removing highly competitive invasive species (Lautenbach et al., 1995). These strategies, however, are only effective if they are accurately matched to limiting factors. For example, implementing seed addition strategies will not be useful if soil environmental conditions act as a strong filter to limit the establishment of focal species. Likewise, removing invasive species from highly fragmented habitats will not benefit species that are dispersal limited.

Elucidating the role of different community assembly processes on biodiversity patterns, however, presents substantial challenges due to both the integrated nature of local and regional effects (Cavender‐Bares et al., 2009; Williams et al., 2009) and the myriad ways in which biodiversity is measured and interpreted (Purvis & Hector, 2000). The term “biodiversity” describes multiple components of diversity, including the richness and abundance of species, functional traits, and genotypes (DeLong, 1996). Biodiversity is often used as a general term, which implies that these different metrics are equivalent. However, taxonomic and functional patterns of diversity are seldom complimentary and often provide different insights into the structure and functioning of ecological communities. For example, several previous studies have shown that general conclusions regarding the assembly and functioning of plant communities are strongly dependent on the metric used to assess biodiversity; that is, species diversity does not necessarily track functional trait changes in a community (Cadotte et al., 2011; Fukami et al., 2005; Mayfield et al., 2005). Thereby suggesting that multiple components of biodiversity must be considered when assessing the general structure and functioning of ecological communities in different environments.

Biodiversity patterns are also not static through time. The dynamic nature of plant communities has been cataloged, studied, and debated for over 100 years (Wiens, 2016). Numerous studies of succession have shown that the relative influence of different assembly processes changes through time, dramatically influencing both taxonomic and functional diversity in unique ways (Kahmen & Poschlod, 2004; Lohbeck et al., 2014; White & Jentsch, 2004). In general, dispersal is expected to play a strong role in early‐successional seres, when suitable habitat is available. Over time, as niches are occupied and resources become limited, competition plays a stronger role in influencing local communities (Aicher et al., 2011). Yet, the field of urban ecology has primarily developed from observational studies of species at a single point in time (Hobbs, 1988; Dallimer et al., 2012; Knapp et al., 2012; but see Johnson et al., 2018; Pyšek et al., 2004). It is thus unclear how native plant species respond to urban environmental conditions over time, and whether these changes influence the long‐term establishment and persistence of native plants in different urban habitats.

Here, we explore the relative influence of local assembly processes on seeded native plant communities over time. Specifically, we experimentally assessed the effects soil environmental filtering (i.e., urban fill soil vs. topsoil) and competition from species that recruited into the experimental plots (i.e., spontaneous urban species) on different dimensions of biodiversity (i.e., taxonomic and functional) over four growing seasons in 2016–2018. It is well‐established that the urban environment consists of a mosaic of habitat patches with highly variable environmental conditions (Machlis et al., 1997; Swan et al., 2021). Conducting an experimental study that includes the full complement of unique urban environments would thus be ideal for gaining generalizable insights into urban plant community assembly patterns; however, such an experimental design is not logistically feasible. We therefore focused on a single land‐use type as a model system for our study, namely urban vacant land. In urban environments, vacant land represents remnants of build infrastructure (e.g., residential housing) that have been demolished and left undeveloped (Pagano & Bowman, 2000). Vacant land is a common feature in most urban landscapes, especially “shrinking cities” (Pallagst et al., 2009) where long‐term urban population losses have left an abundance of unused land parcels. For example, in Baltimore City, a loss of nearly 400,000 residents since the 1960s (U.S. Census Bureau, 2018) has resulted in over 25,000 vacant lots, and another 17,000 abandoned houses, which are slated to be razed in the coming years (McHugh, 2012). Once a building has been demolished and the area leveled using imported subsoil fill material, urban vacant land typically persists under minimal management, allowing plant communities to establish naturally. As a result, these areas often contribute substantially to urban biodiversity by serving as derelict habitats for an array of species (Muratet et al., 2007). This characteristic makes urban vacant land a potential asset for urban regreening efforts aimed to enhance biodiversity and ecosystem services in cities (Burkholder, 2012). By focusing on a single, minimally managed urban habitat type, we were also able to more effectively assess the filtering effects of local assembly processes and avoid confounding factors associated with past and present human‐management pressures (Vallet et al., 2010).

In this study, we expected taxonomic and functional diversity patterns to differ across treatment groups and time (H1). Specifically, we expected Shannon diversity to be strongly influenced by competition from spontaneous urban species exhibited by a decrease in Shannon diversity in unweeded plots than in weeded plots. By contrast, we expected soil environmental filtering would have a greater effect on functional diversity over time exhibited by a decrease in functional diversity in urban fill than in topsoil. We also expected compositional changes to vary according to the relative influence of different local assembly processes (H2). Namely, if soil environmental filtering has a strong effect on the composition of native species, we predict both taxonomic and functional composition (i.e., the multivariate distribution of species or functional trait values, respectively, in a community) will be lower in urban, subsoil fill material compared with topsoil, as harsh edaphic conditions associated with urban fill are expected to limit the establishment of species, as well as the abundance and range of their functional trait values (Cornwell & Ackerly, 2009). Likewise, if spontaneous urban species exhibit strong competitive effects on natives, we predict taxonomic and functional composition will be higher in weeded plots where competitive pressures are relaxed. Finally, we expected species diversity and composition to shift over time (H3), as later‐successional species, with different functional strategies, replace early‐successional species (Chang & Turner, 2019; Lososová et al., 2006; Schadek et al., 2008).

## 2 METHODS

### 2.1 Study design

**FIGURE 1:**
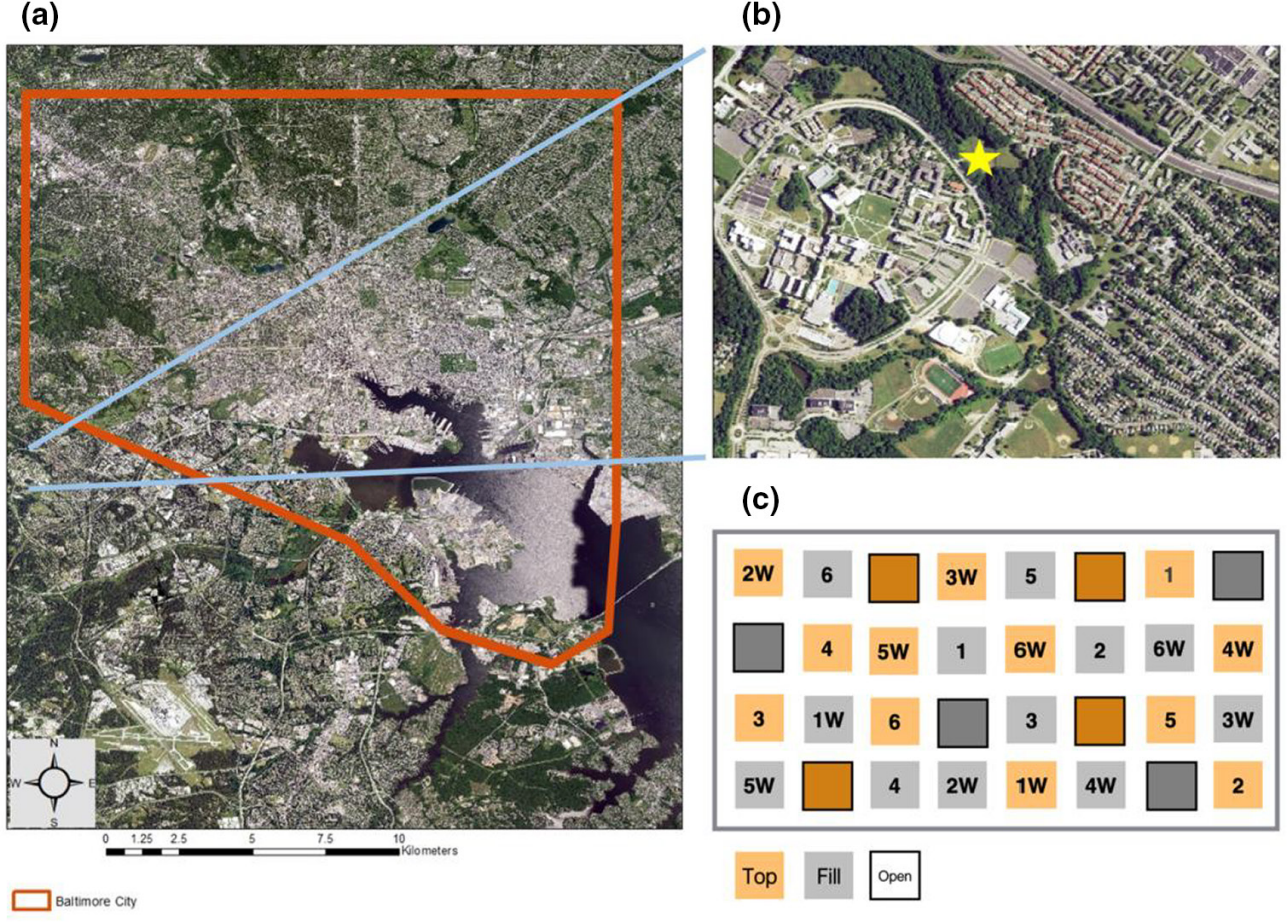
(a) Map of study site location relative to Baltimore City, Maryland (orange outline) and (b) location on the University of Maryland, Baltimore County campus (star). (c) Illustration of the experimental plot layout. Soil treatment groups are indicated by color (orange‐topsoil; gray‐fill) and competition treatment groups are indicated by a “W” for weeded and no letter for unweeded plots; solid lines around boxes indicate control groups that were not seeded, and numbers in each box indicate the seeded native “community type.”

To address these questions, we designed a common garden study consisting of 32, 2‐m^2^ raised experimental plots, which were separated by a 2‐m wide aisle covered in landscape fabric, located on the University of Maryland, Baltimore County campus (39°15′N, 76°42′W, 75 m; Figure 1). Replicated treatments (6x each) were crossed in a 2 x 2 (urban fill vs. screened topsoil, weeded vs. unweeded) full‐factorial randomized design. Eight plots (four urban fill and four topsoil) acted as open, control treatments and were not seeded with a native species seed mix. The “urban soil” treatment was selected to simulate vacant land soil conditions postdemolition. Subsoil fill material was sourced directly from a company contracted for vacant housing demolition projects in Baltimore City, MD (M.R. Dirt, Towanda, PA). Screened topsoil was sourced locally to replicate typical soil conditions for native species used in the study. These soil treatments do not account for the full range of possible soil types found in undisturbed sites or urban vacant land (Herrmann et al., 2017; Pickett et al., 2011; Pouyat et al., 2007). They are, however, the most common soil types in anthropogenic environments (Gilbert, 1989), and exemplify the opposing conditions in terms of potentially strong (i.e., urban fill) and weak (i.e., topsoil) environmental filters.

In our study, weeding treatments isolate interspecific competition from urban plant species that have either recruited into the plots from the surrounding region or that were present in the soil seed bank (i.e., spontaneous species). All species, both native and non‐native, that were not seeded were removed from the weeded treatment plots. Plots were weeded monthly during the growing season (May–August) and prior to data collection in November and July 2016–2017; tree seedlings were concomitantly removed from all plots. All spontaneous species that recruited into the study plots have been surveyed in urban vacant lots in Baltimore City (Johnson et al., 2015, 2018), indicating that the regional species pool is consistent across study sites. We seeded all treatment plots, except open control plots, with one of six native “community types,” which consisted of 10 herbaceous plant species randomly drawn from a pool of 25 species native to Maryland. We avoided species with narrow habitat ranges or specialists, such as wetland or obligate forest understory species, due to the likelihood that they would not germinate or survive in our experimental plots. We did, however, include several species that are associated with forest and woodland habitats, namely *Thalictrum thalictroides* and *Oxalis violacea*. In addition, each native community was composed of species representing different growth forms, life history strategies, and dispersal modes (Table S1). This design captured variation in community composition and ensured that each community represented a diverse array of functional groups, which limiting bias associated with community type. To minimize genotypic variation across ecotones (Joshi et al., 2001), we sourced all seeds from commercial producers that collect and propagate seeds of Northeastern regional provenances (Ernst Conservation Seeds, Inc., PA; Prairie Moon, MN; Chesapeake natives, MD). We followed a substitutive design for all seed additions; whereby, the total density of seeds was kept equal across plots, but the seed density for each species in a community was adjusted for seed mass‐seed number trade‐offs (Murray et al., 2005; Rees, 1995; Westoby et al., 1992; i.e., we added lower densities of large seeds and higher densities of small seeds to each community). Total seed density was set at 2.5 g/m^2^, which is comparable with other seed addition studies (e.g., Aicher et al., 2011; Hitchmough et al., 2004; Myers & Harms, 2009) and the recommended rate for meadow restoration (Diboll, n.d.). We sowed seeds in October 2015 and 2016 to allow them to naturally stratify.

### 2.2 Sampling design and species surveys

We sampled seeded native and spontaneous plant communities biannually during peak growth and peak standing biomass (mid‐July and early November, respectively) beginning from November 2016 to June 2018 (Figure S1). We used a modified Braun‐Blanquet relevé method to visually estimate percentage cover of each species (Braun‐Blanquet, 1932), using a 1‐m^2^ quadrat placed in the center of each plot, to avoid edge effects. We identified all plants to the species level and estimated percent cover using midpoint values of seven cover classes (i.e., <1% was estimated as 0.005%, 1%–5% as 3%, 6%–10% as 7.5%, 11%–25% as 17.5%, 26%–50% as 37.5%, 51%–75% as 62.5%, and 76%–100% as 87.5%).

### 2.3 Functional trait measures

We selected seven traits that represent key axes of plant ecological strategies related to dispersal capacity, establishment, and persistence (Weiher et al., 1999). They included continuous traits, that is, average seed weight, maximum vegetative height (H_max_), specific leaf area (SLA), and leaf chlorophyll *a* fluorescence (Chl *a*), as well as categorical traits related to biophysical characteristics, that is, photosynthetic pathway (C3 graminoid and C4 graminoid), growth form (forb and legume), and life form (annual and biannual/perennial). Seed weight is associated with seedling fitness and resource allocation trade‐offs (i.e., seed size‐seed number and competition‐colonization trade‐offs, Jacobsson & Eriksson, 2000; Turnbull et al., 2004). It serves as a proxy for both spatial, that is, the potential distance a seed can move, and temporal, that is, seed bank retention time, dispersal potential, with smaller seeds generally having long‐distance dispersal capacities and a more persistent seed bank, which allows them to remain viable in soil for longer periods of time, compared with larger seeds (Cornelissen et al., 2003). Seed weight is expected to increase with plant height and under drier and warmer conditions (Pakeman et al., 2008). Height is a persistence trait that is associated with competitive vigor and relative growth rate (Cornelissen et al., 2003; Weiher et al., 1999; Westoby, 1998). SLA is part of the “leaf economics spectrum,” which describes a broad range of ecological strategies related to establishment and persistence, including photosynthetic capacity, resource acquisition, and water use efficiency (Westoby et al., 2002; Wright et al., 2004). Finally, Chl *a* is a biochemical trait that relates to photochemical changes associated with plant stress, with lower values corresponding to increased water and/or nutrient stress (Schreiber & Bilger, 1987).

For all trait measures, we sampled three mature individuals of each represented species in each plot. Trait measures were taken concomitantly with species surveys, except Chl *a*, which was not measured in the first season because the Chl *a* fluorescence equipment was unavailable. We followed standard protocols for all trait measures (Cornelissen et al., 2003; Pérez‐Harguindeguy et al., 2013). All seed and leaf samples were oven‐dried at 70°C for a minimum of 72 h prior to weighing. We calculated seed weight by collecting and weighing a known number of ovendried seeds for each individual and dividing the total weight by the seed number. Seeds with visible protective structures or attachments (e.g., wings, burs, and plumes) were weighed whole. We measured plant height as the distance (in cm) between the ground and the highest photosynthetic tissue or “stretch length” for recumbent species. SLA is a composite measure that is calculated by dividing the total area of a leaf by its dry mass. For each sampled individual, we measured SLA on three fully expanded, undamaged leaves, including petiole. We measured leaf area directly in the field using a LI‐3000C Portable Leaf Area Meter (Li‐Cor, Lincon, NE). We measured Chl *a* fluorescence using a Chlorophyll Content Meter Model CCM‐330 (Opti‐sciences, Jackson, MS) on the same leaves sampled for SLA, prior to removal.

### 2.4 Soil data

We collected soil samples in November 2016 (hereafter, season 1) and June 2018 (hereafter, season 4) from all study plots, including two reference samples of fill and topsoil that were not used in the study. Soil samples were sent to the Cornell Nutrient Analysis Laboratory (Ithaca, NY) for analysis of micro‐ and macronutrient concentrations, pH, organic matter (LOI), and heavy metal concentrations using a modified Mehlich‐I extraction technique (Burt, 2004). This analysis technique measures a variety of soil nutrients and abiotic characteristics that have been used in prior studies to characterize urban soils (Johnson et al., 2015; Pavao‐Zuckerman, 2008; Pouyat et al., 2010). Notably, this technique cannot reliably measure available nitrogen, due to its volatility in the environment; therefore, nitrogen compounds (i.e., ammonium and nitrate) were not included in the soil data. Because our study plots were only 18″ deep, we collected soil samples via a hand trowel from the corners (6–8″ away from the plot border) and the center of each plot, for a total of five samples per plot. We composite mixed and air‐dried soil samples from each plot for seven days prior to shipping. For the soil analysis, we selected standard soil variables that are essential for plant growth and development, as well as variables that are common contaminants in urban environments, which can negatively impact plant survival. These included the following: % moisture, pH, organic matter (OM), aluminum (Al), arsenic (As), calcium (Ca), cadmium (Cd), chromium (Cr), copper (Cu), iron (Fe), potassium (K), magnesium (Mg), manganese (Mn), sodium (Na), phosphorus (P), lead (Pb), and Zinc (Zn).

### 2.5 Statistical analyses

In total, we included four sample seasons in our analyses (season 1: November 2016; season 2: June 2017; season 3: November 2017; and season 4: June 2018). All taxonomic and functional diversity analyses were in implemented in R (v 3.3.3, R R Core Team, 2017) and were based on species' relative percent cover estimates. To estimate taxonomic diversity, we calculated the Shannon diversity index (H) for each plot treatment group and seeded community type. Prior to estimating functional diversity, we log_10_‐transformed all continuous traits (i.e., H_max_, SLA, seed weight, and Chl *a*) to normalize the data and then range‐standardized traits using the “decostand” function in the R *vegan* package (v 2.4–3, Oksanen et al., 2017). We then estimated functional diversity using the functional dispersion (FDis) index, which calculates the abundance‐weighted mean distance of species to the community centroid in multidimensional trait space (Laliberté & Legendre, 2010). This index estimates functional alpha diversity and is suitable for ecological data because it is not affected by species richness or outliers, it can accommodate different types of traits, as well as any distance metric and number of traits—including scenarios where traits outnumber species (Laliberté & Legendre, 2010). FDis of traits was calculated using Gower's distance, as it can accommodate missing values and mixed variables (de Bello et al., 2013).

We tested for differences in taxonomic and functional diversity across treatment groups and seasons using mixed‐effects ANOVA models. We fitted five models and selected the most parsimonious model based on significance (*α* = 0.05) and Akaike information criterion (AIC). All models, except models 1 and 5 (see below), incorporated soil, competition, season, and their interaction as fixed effects. Models included (1) a baseline random intercept model; (2) a model with no random effects; (3) a model incorporating community type as a random effect; (4) a model incorporating community type as a random effect and a compound symmetry correlation structure—using the “corCompSym” function in the *nlme* package (v 3.1–131, Pinheiro et al., 2017)—to account for repeated measures across seasons; and (5) a model incorporating community type nested within season as a random effect with a compound symmetry correlation structure. The model with the lowest AIC consistently included soil (two levels: fill and topsoil), competition (two levels: weeded and unweeded), season (four levels: season 1‐season 4) and their interaction (soil x competition × season) as fixed effects, and community type (six levels) as a random effect (i.e., model 3). We conducted post hoc Tukey's HSD tests to evaluate pairwise differences across each model group.

To assess changes in taxonomic and functional composition across seasons and experimental plots with different soil and weeding treatments, we performed permutational multivariate ANOVA (PERMANOVA; Anderson, 2001) based on pairwise distance matrices, using the “adonis” function in the *vegan* R package (v 2.4–3, Oksanen et al., 2017). We measured taxonomic dissimilarity across seasons and treatment groups using the Bray–Curtis distance metric, and Gower's distance for determining functional dissimilarity. We tested the PERMANOVA assumption of multivariate homogeneity of group dispersions using the “betadisper” function (Oksanen et al., 2017). Because continuous trait measures were taken directly on multiple individuals in each experimental plot, versus collated from databases as species‐level estimates, we averaged trait values across individuals for each species at the plot‐level (versus the species‐level) and used these values in our compositional and CWM analyses. We conducted pairwise comparisons of significant PERMANOVA factors using the “pairwise.perm.manova” function in the *RVAideMemoire* R package (v 0.9–66, Hervé, 2017) with the “fdr” adjustment method for multiple comparisons (Benjamini & Hochberg, 1995; Benjamini & Yekutieli, 2001), which is appropriate for repeated measures as it corrects for the false discovery rate. We calculated community‐weighted mean (CWM) of each functional trait, which represents the average trait values in a community of species weighted by their relative abundance (Díaz et al., 2007; Garnier et al., 2004; Lepš et al., 2011), in order to compare changes in each trait across seasons, soil, and competition treatment groups.

We assessed patterns in soil parameters for urban fill and topsoil in seasons 1 (November 2016) and 4 (June 2018) using principal components analysis (PCA). All PCAs were conducted in the *FactoMineR* package in R (Lê et al., 2008), and PCAs were calculated with the *PCA* function. To accommodate comparisons across magnitudes of variation and different units of measurement, we range‐standardized soil variables to scale to a mean of zero and a variance of one, prior to analysis (Table S2). Finally, we calculated the correlation coefficient and associated *p*‐values between each soil variable and dimension using the “dimdesc” function. Only soil variables with significant correlations (α = 0.05) were retained as vectors for the PCA biplot.

## 3 RESULTS

Of our seeded pool, four species did not successfully germinate and establish (Table S1). We attribute this to strong environmental filtering effects that excluded natives with specific habitat requirements. In particular, all unrepresented natives prefer partial sun exposure and moist‐mesic soil conditions, and several species grow primarily in forest understory habitats. By contrast, our study area was located in a clearing with full sun exposure. Additionally, the limited size and raised position of our study plots meant that soil moisture was highly variable, that is, soil conditions changed from saturated to dry over relatively short periods. One species, *Elymus hystrix* L., only appeared later in our study (i.e., seasons 3 and 4), which may indicate that this species has a longer seed dormancy period than the other native species in our pool.

### 3.1 Taxonomic and functional diversity

**FIGURE 2:**
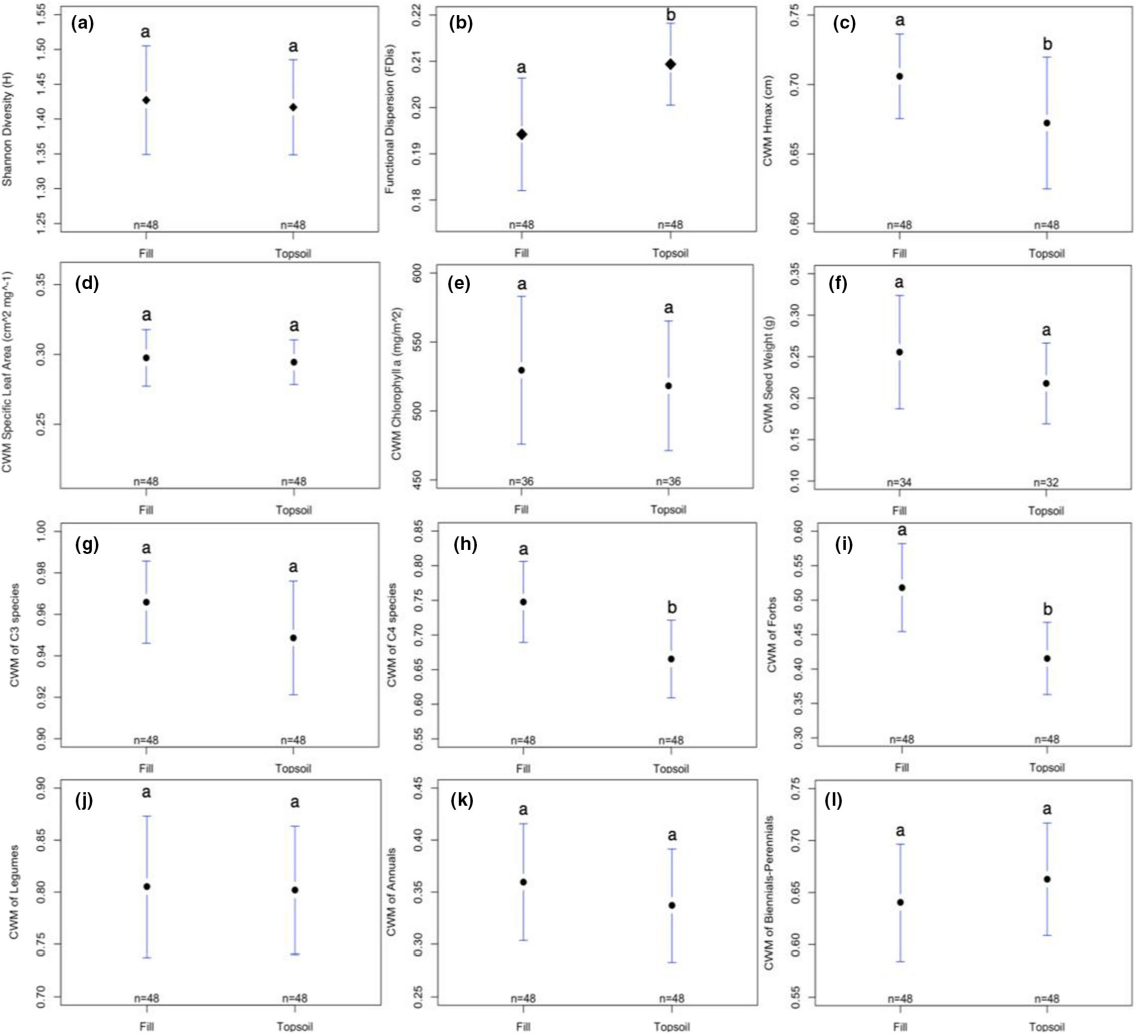
Plotmeans of (a) taxonomic diversity (Shannon H), (b) functional diversity (FDis), (c) CWM Hmax, (d) CWM specific leaf area (SLA), (e) CWM chlorophyll *a*, (f) CWM seed weight, (g) CWM C3 species, (h) CWM C4 species, (i) CWM forbs, (j) CWM legumes, (k) CWM annuals, (l) CWM biennials/perennials with 95% CIs for soil type (fill vs. topsoil). Letters indicate significant differences between pairwise comparisons of seasons (*p* ≤ .05).

**FIGURE 3:**
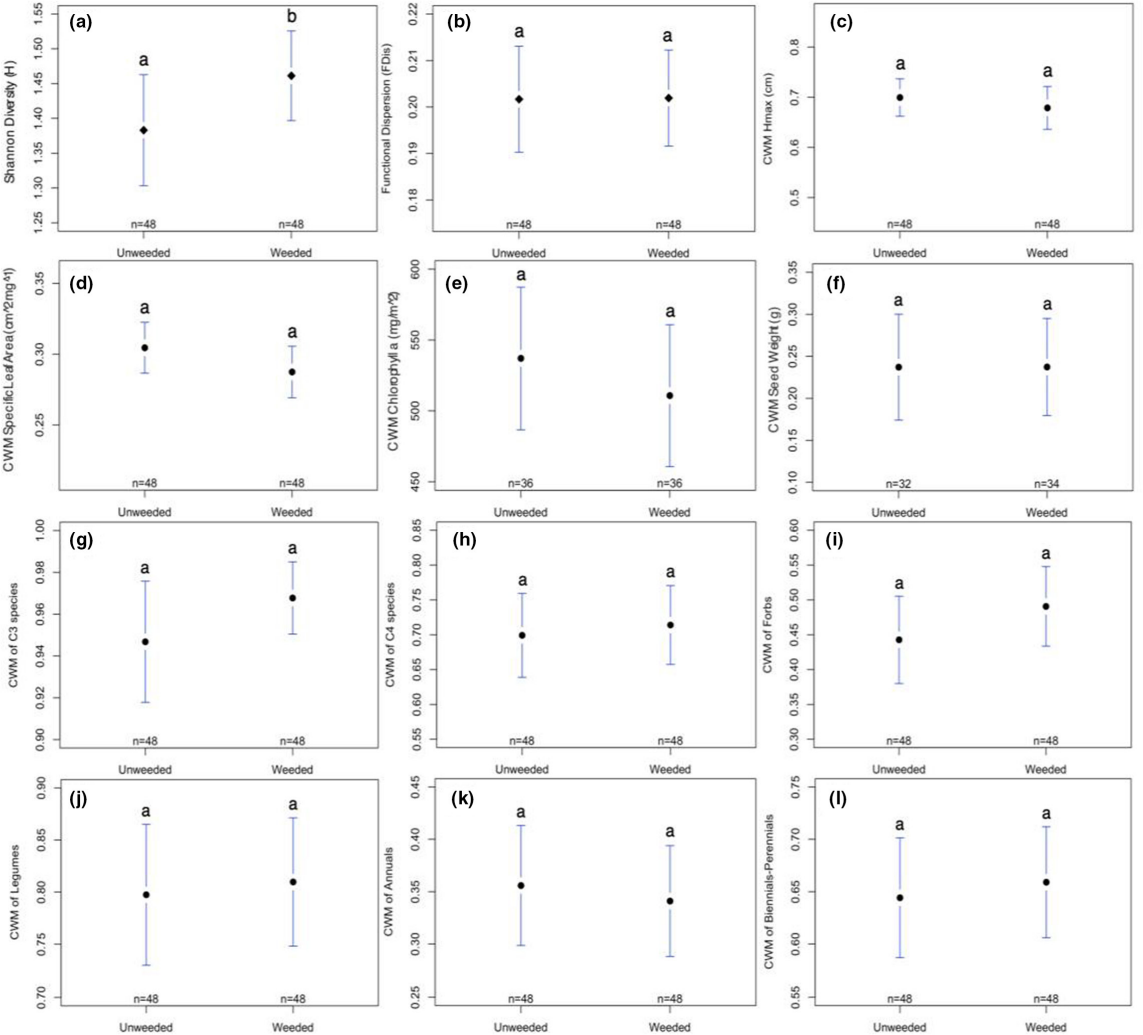
Plotmeans of (a) taxonomic diversity (Shannon H), (b) functional diversity (FDis), (c) CWM Hmax, (d) CWM specific leaf area (SLA), (e) CWM chlorophyll *a*, (f) CWM seed weight, (g) CWM C3 species, (h) CWM C4 species, (i) CWM forbs, (j) CWM legumes, (k) CWM annuals, (l) CWM biennials/perennials with 95% CIs for competition treatment groups (unweeded vs. weeded). Letters indicate significant differences between pairwise comparisons of seasons (*p* ≤ .05).

**FIGURE 4:**
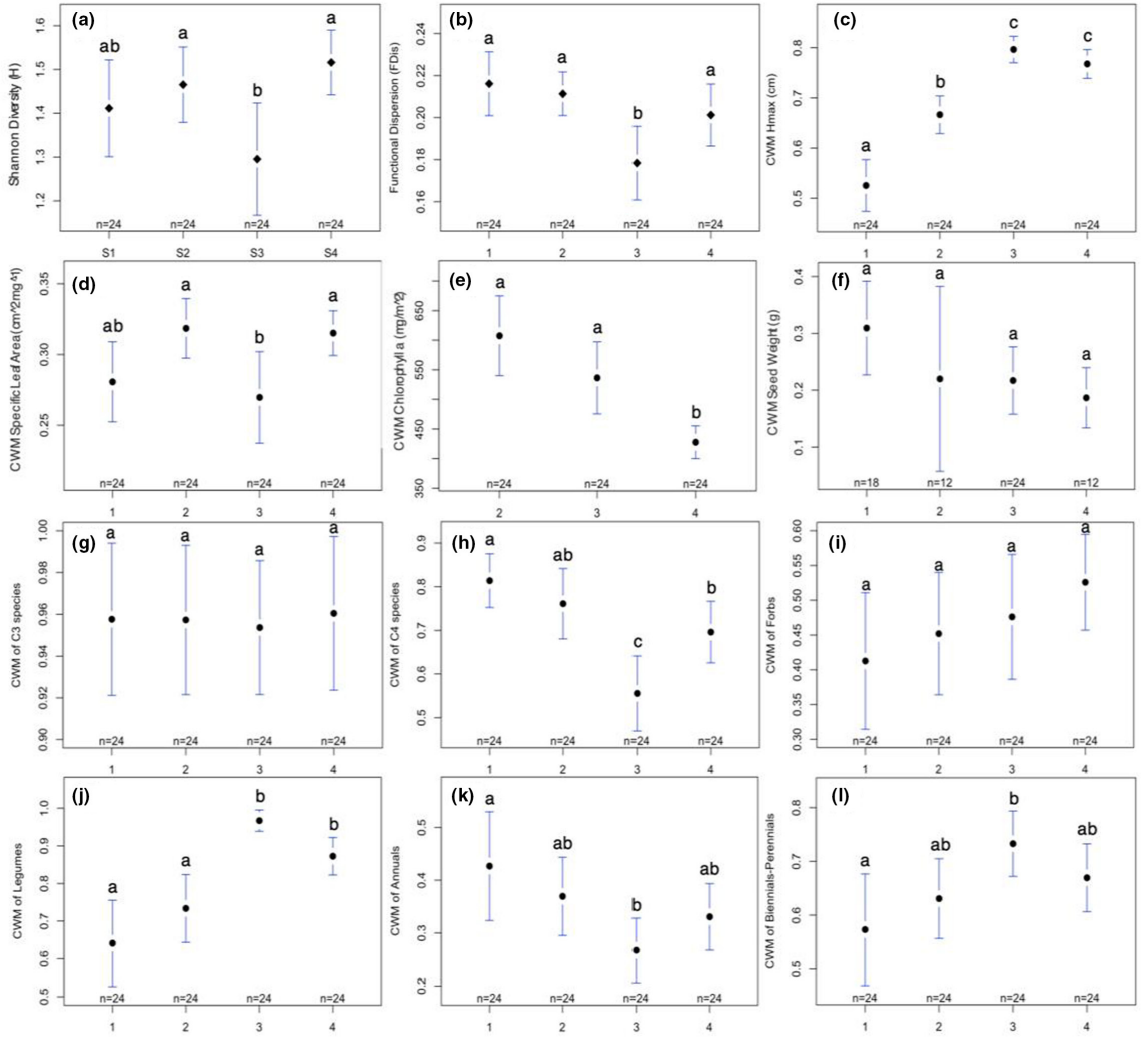
Plotmeans of (a) taxonomic diversity (Shannon H), (b) functional diversity (FDis), (c) CWM Hmax, (d) CWM specific leaf area (SLA), (e) CWM chlorophyll *a*, (f) CWM seed weight, (g) CWM C3 species, (h) CWM C4 species, (i) CWM forbs, (j) CWM legumes, (k) CWM annuals, (l) CWM biennials/perennials with 95% CIs for season treatment groups (years 1–4). Letters indicate significant differences between pairwise comparisons of seasons (*p* ≤ .05).

Soil did not have an effect on native taxonomic diversity (Table 1, Figure 2a); however, both competition and season significantly influenced native taxonomic diversity (Table 1, Figures 3a and 4a, respectively). Post hoc pairwise comparisons showed that Shannon diversity was higher in weeded vs. unweeded plots (*t*_75_ = −2.09, *p* = .04) and was lower in season 3 than in seasons 2 (*t*_75_ = 3.21, *p* = .01) and 4 (*t*_75_ = −4.17, *p* = .0005). Comparisons between all other seasons were nonsignificant (*p* > .05).

**TABLE 1:** Mixed‐effects ANOVA of the of the effect of soil (fill vs. topsoil), competition (weeded vs. unweeded), season (S1–S4), and interactions (soil × competition, soil × season, competition × season, soil × competition × season) on taxonomic diversity (Shannon H), functional diversity (FDis), and community‐weighted mean of traits.

	Variable	df	*F*	*p*
Shannon H	Soil	1, 75	0.07	.787
	Competition	1, 75	4.37	**.040**
	Season	3, 75	6.40	**.001**
	Soil × Competition	1, 75	3.01	.087
	Soil × Season	3, 75	0.37	.778
	Competition × Season	3, 75	0.49	.692
	Soil × Competition × Season	3, 75	1.30	.281
FDis	Soil	1, 75	5.31	**.024**
	Competition	1, 75	0.002	.970
	Season	3, 75	6.50	**.0006**
	Soil × Competition	1, 75	1.68	.199
	Soil × Season	3, 75	0.20	.894
	Competition × Season	3, 75	0.09	.964
	Soil × Competition × Season	3, 75	0.91	.439

*Note*: Significant differences (*p* ≤ .05) denoted in bold.

When comparing functional diversity metrics, we found no effect of competition (Table 1, Figure 3b); however, functional dispersion (FDis) differed significantly between soil types and across seasons (Table 1, Figures 2b and 4b, respectively). Post hoc analyses indicated that FDis was greater in topsoil treatments than fill (*t*_75_ = −2.30, *p* = .024), and, similar to Shannon diversity, was lower in season 3 than all other seasons (seasons 1–3: *t*_75_ = 4.06, *p* = .0007; 2–3: *t*_75_ = 3.54, *p* = .004; 3–4: *t*_75_ = −2.45, *p* = .076).

### 3.2 Compositional divergence among treatment groups and species trait distributions

**FIGURE 5:**
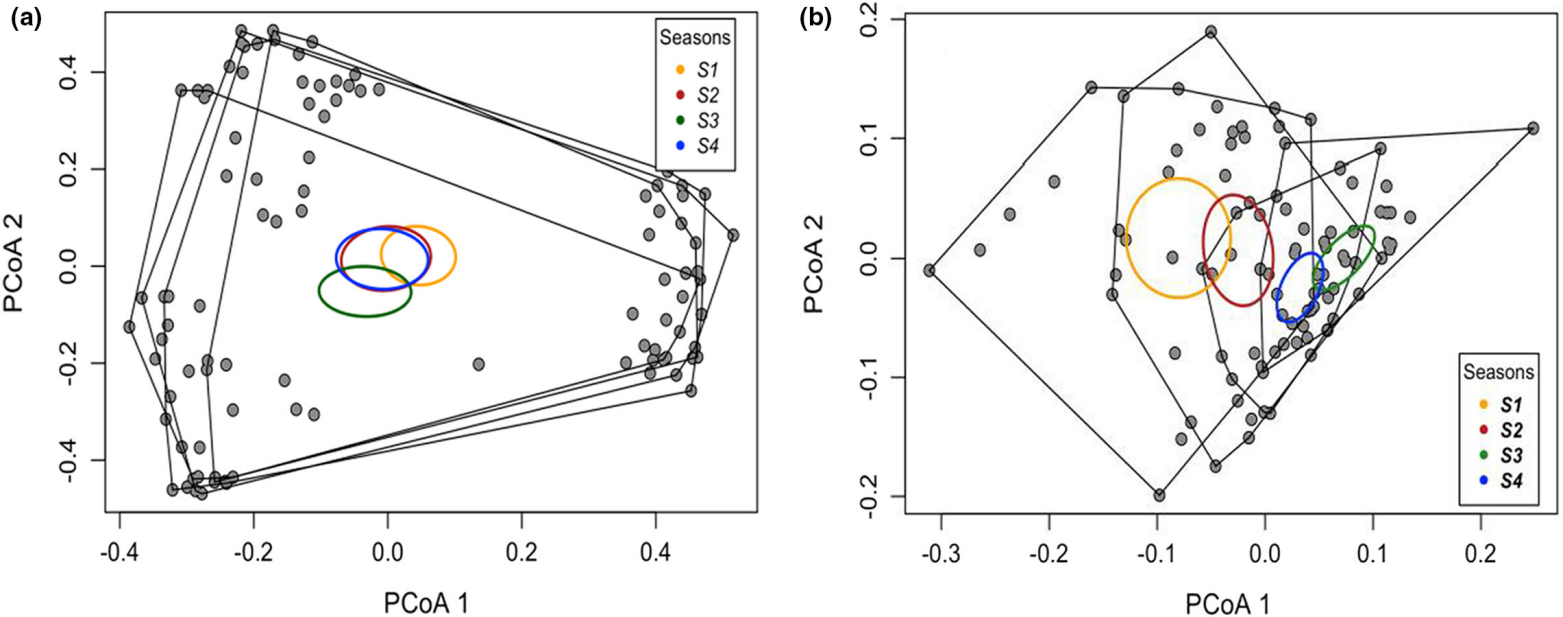
Principal coordinate analysis (PCoA) of (a) taxonomic composition and (b) functional composition data (*n* = 24 for each season) with convex hulls differentiating each season and 95% confidence ellipses.

We found no significant divergence in taxonomic or functional composition between soil types, competition treatment groups, or any interactions (*p* > .05; Table 2). Across seasons, however, taxonomic composition did significantly diverge (Table 2, Figure 5a); pairwise permutational comparisons indicated that composition differed in early seasons, but did not change between later seasons (seasons 1–2: *p* = .078; 1–3: *p* = .003; 1–4: *p* = .003; 2–3: *p* = .028; 2–4: *p* = .149; 3–4: *p* = .162). Functional composition also significantly diverged across seasons (Table 2, Figure 5b), and post hoc pairwise comparisons showed that functional composition shifted between all seasons (seasons 1–2: *p* = .004; 1–3: *p* = .001; 1–4: *p* = .001; 2–3: *p* = .001; 2–4: *p* = .001; 3–4: *p* = .001). Comparing CWM values of individual functional traits, we found significant differences in the distribution of all traits except seed weight and the proportion of C3 grasses (Table 2, Figures 2, 3, and 4c–l). Differences in CWMs were most commonly observed across seasons (either individually or as interactions); although, H_max_ also differed between soil treatments (Table 2, Figure 2c), the proportion of C4 grasses differed between soil treatments and competition × soil interaction (Table 2, Figure 2h), and the proportion of forbs differed only between soil types (Table 2, Figure 2i). Competition from spontaneous species did not significantly influence CWM trait values for any measured trait (Figure 3c–l).

**TABLE 2:** PERMANOVA of the effect of soil (fill vs. topsoil), competition (weeded vs. unweeded), season (S1–S4), and interactions (soil × competition, soil × season, competition × season, soil × competition × season) on taxonomic and functional composition

	Variable	df	*F*	*p*
Taxonomic composition	Soil	1, 80	0.95	.457
	Competition	1, 80	0.42	.874
	Season	3, 80	3.19	**.001**
	Soil × Competition	1, 80	0.50	.804
	Soil × Season	3, 80	0.26	.999
	Competition × Season	3, 80	0.30	.996
	Soil × Competition × Season	3, 80	0.15	1.000
Functional composition	Soil	1, 80	1.33	.126
	Competition	1, 80	0.84	.654
	Season	3, 80	4.19	**.001**
	Soil × Competition	1, 80	0.98	.457
	Soil × Season	3, 80	0.97	.495
	Competition × Season	3, 80	0.72	.982
	Soil × Competition × Season	3, 80	0.82	.843
CWM H_max_	Soil	1, 75	6.17	**.015**
	Competition	1, 75	2.32	.132
	Season	3, 75	81.84	**<.0001**
	Soil × Competition	1, 75	1.83	.180
	Soil × Season	3, 75	6.34	**.0007**
	Competition × Season	3, 75	0.70	.553
	Soil × Competition × Season	3, 75	0.72	.541
CWM SLA	Soil	1, 75	0.07	.787
	Competition	1, 75	2.22	.141
	Season	3, 75	4.56	**.006**
	Soil × Competition	1, 75	0.01	.904
	Soil × Season	3, 75	0.58	.631
	Competition × Season	3, 75	1.22	.308
	Soil × Competition × Season	3, 75	0.68	.565
CWM seed weight	Soil	1, 45	0.80	.375
	Competition	1, 45	0.00	.997
	Season	3, 45	1.51	.224
	Soil × Competition	1, 45	0.02	.895
	Soil × Season	3, 45	0.78	.510
	Competition × Season	3, 45	0.97	.414
	Soil × Competition × Season	3, 45	0.88	.457
CWM Chl *a*	Soil	1, 55	0.14	.706
	Competition	1, 55	0.78	.381
	Season	2, 55	12.36	**<.0001**
	Soil × Competition	1, 55	2.11	.152
	Soil × Season	2, 55	0.04	.964
	Competition × Season	2, 55	0.03	.967
	Soil × Competition × Season	2, 55	1.75	.183
CWM C3 grass	Soil	1, 75	1.56	.216
	Competition	1, 75	2.30	.134
	Season	3, 75	0.04	.989
	Soil × Competition	1, 75	0.07	.795
	Soil × Season	3, 75	1.95	.129
	Competition × Season	3, 75	0.76	.519
	Soil × Competition × Season	3, 75	1.75	.164
CWM C4 grass	Soil	1, 75	13.71	**.0004**
	Competition	1, 75	0.45	.506
	Season	3, 75	25.24	**<.0001**
	Soil × Competition	1, 75	6.75	**.011**
	Soil × Season	3, 75	1.41	.248
	Competition × Season	3, 75	1.10	.355
	Soil × Competition × Season	3, 75	0.66	.577
CWM forbs	Soil	1, 75	8.26	**.005**
	Competition	1, 75	1.80	.184
	Season	3, 75	1.75	.164
	Soil × Competition	1, 75	1.00	.321
	Soil × Season	3, 75	1.34	.267
	Competition × Season	3, 75	0.54	.659
	Soil × Competition × Season	3, 75	0.49	.688
CWM legumes	Soil	1, 75	0.01	.914
	Competition	1, 75	0.16	.693
	Season	3, 75	21.85	**<.0001**
	Soil × Competition	1, 75	0.35	.556
	Soil × Season	3, 75	0.07	.975
	Competition × Season	3, 75	0.31	.818
	Soil × Competition × Season	3, 75	0.26	.854
CWM annual	Soil	1, 75	0.50	.480
	Competition	1, 75	0.22	.639
	Season	3, 75	4.55	**.006**
	Soil × Competition	1, 75	1.15	.286
	Soil × Season	3, 75	1.68	.178
	Competition × Season	3, 75	0.04	.987
	Soil × Competition × Season	3, 75	0.78	.511
CWM biennial‐perennial	Soil	1, 75	0.50	.480
	Competition	1, 75	0.22	.639
	Season	3, 75	4.55	**.006**
	Soil × Competition	1, 75	1.15	.286
	Soil × Season	3, 75	1.68	.178
	Competition × Season	3, 75	0.04	.987
	Soil × Competition × Season	3, 75	0.78	.511

*Note*: Significant differences (*p* ≤ .05) denoted in bold.

### 3.3 Comparison of soil characteristics

**FIGURE 6:**
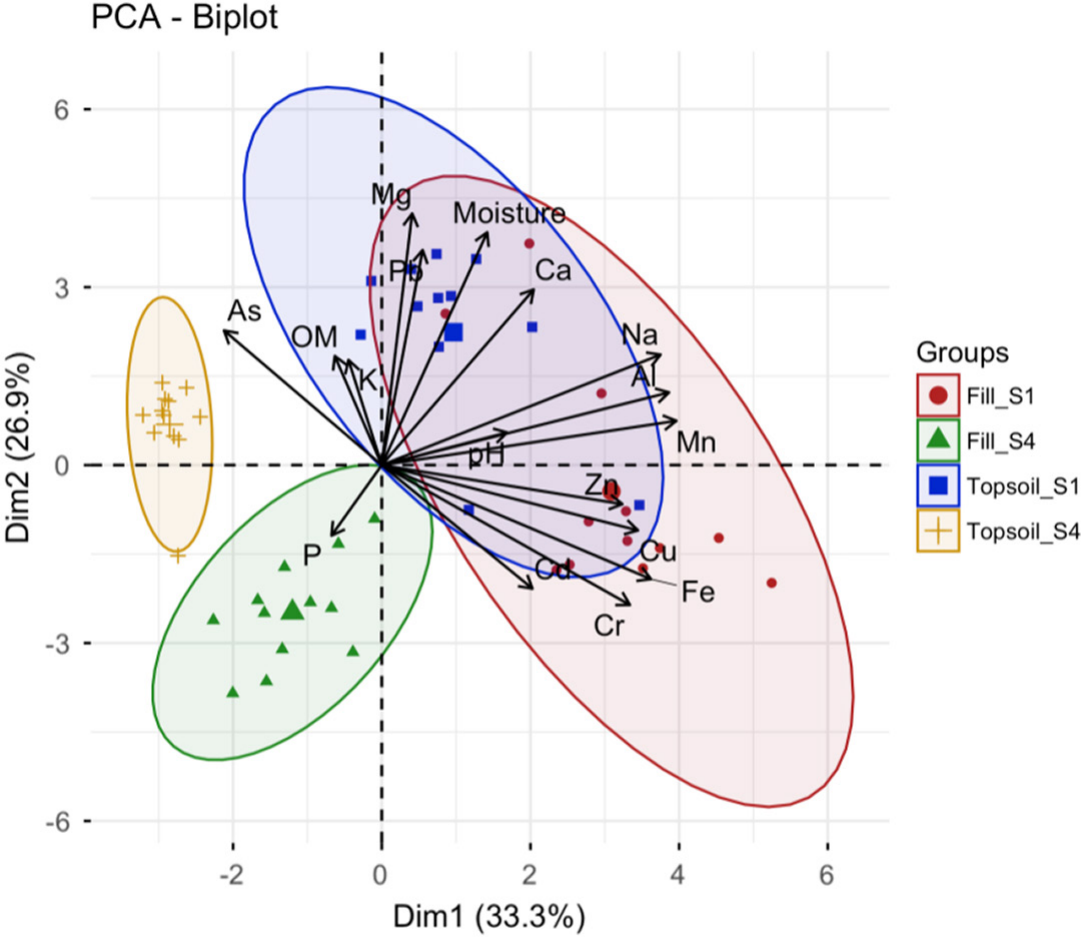
PCA biplot of soil variables for urban fill and topsoil treatments measured in seasons 1 (S1) and 4 (S4). Soil variables include % moisture, pH, organic matter (OM), aluminum (Al), arsenic (as), calcium (ca), cadmium (cd), chromium (Cr), copper (cu), iron (Fe), potassium (K), magnesium (mg), manganese (Mn), sodium (Na), phosphorus (P), lead (pb), zinc (Zn). Only soil variables that were significantly correlated with the first two principal component axes (*p* ≤ .05) were retained as vectors for the biplot.

In total, we analyzed 70 soil samples, and report results for each soil treatment group sampled in each season (Table S2). Reference soil samples did not differ from season 1 samples and were thus not included in our results. The PCA ordination plot showed that soil correlations were relatively well captured by the first two axes; variance explained by the first and second axes was 33.3% and 26.9%, respectively (Figure 6). Overall, the first axis was determined primarily by soil heavy metal concentrations (Mn, Al, Fe, Cu, Cr, Zn, Cd, and As), Na, and pH, whereas soil moisture, OM, Pb, and macronutrient concentrations (Ca, Mg, and K) were strongly positively correlated with the second axis (Table S3). Fill and topsoil samples strongly diverged over time, both within and between treatment groups. In season 1, fill and topsoil samples showed substantial overlap in soil parameters. By season 4, however, both treatment groups became strongly divergent, with each soil type exhibiting distinct characteristics.

## 4 DISCUSSION

### 4.1 The relative effects of local assembly processes on taxonomic and functional diversity patterns over time

The influence of soil and competition from spontaneous urban species had different effects on the taxonomic and functional diversity of native species, which supports our hypothesis (H1). Overall, we found higher Shannon diversity in plots where spontaneous species were weeded compared with unweeded plots. By contrast, functional diversity (FDis) was not influenced by competition, but did change in response to soil; whereby, topsoil plots exhibited higher functional diversity of natives than urban fill plots. This supports the general conclusion that harsh edaphic conditions associated with urban soils constrain the range of expressed functional trait values, resulting in lower functional diversity of the community. Our results also correspond with prior studies that found weak associations between local taxonomic and functional diversity patterns (Purschke et al., 2013; Johnson et al., 2015; but see Petchey & Gaston, 2002). This is particularly evident when considering continuous traits that exhibit a high potential for phenotypic plasticity such as SLA, height, and chlorophyll *a* (Gratani, 2014 and references therein). High degrees of intraspecific trait variation can decouple taxonomic and functional relationships, such that local environmental conditions and competition can influence functional trait expression without affecting species diversity (i.e., low turnover; Petchey & Gaston, 2006). Conversely, the distribution of functional traits in a community can converge in response to local filtering effects (i.e., environmental constraints or competition, Mayfield & Levine, 2010) despite high rates of species turnover or differences in initial species composition (Fukami et al., 2005). Overall, these results further suggest that taxonomic and functional diversity metrics provide different sources of information for exploring community dynamics and must be carefully considered when developing general conclusions about community structure and functioning (Mayfield et al., 2010).

### 4.2 Seasonal relationships between taxonomic and functional composition

Soil and competition did not affect either taxonomic or functional composition of seeded native species, indicating that the structure and functioning of native species in urban vacant habitats are not substantially influenced by local filtering effects (i.e., soil or competition from spontaneous urban species). These results support our hypothesis (H2) and contradict prior assumptions regarding the low/decreasing proportions of native species in urban environments (Aronson et al., 2014; Kowarik, 1995; Pyšek, 1993). It also suggests that regional factors, such as dispersal limitation, may be the primary driver of community assembly patterns in urban environments. A global meta‐analysis of intra‐urban biodiversity by Beninde et al. (2015) made similar conclusions, finding that factors indirectly associated with dispersal potential, that is, large patch size (> 50 ha) and corridor network, were most important for maintaining high levels of urban biodiversity among different taxa. By contrast, Thompson and McCarthy (2008) found that dispersal traits (i.e., dispersal mode and seed bank persistence) were not reliable determinants of native and alien plant species success along an urban–rural gradient. Such discrepancies among studies suggest that research focusing expressly on seed dispersal pathways is needed to determine exactly how urbanization influences the potential for native species to colonize different urban habitats.

Elucidating the factors that influence the structure and functioning of native plant communities has important implications for urban regreening and sustainability strategies, which in recent years have become focal goals of many cities, including Baltimore (Baltimore Office of Sustainability, n.d.). According to our results, native species are capable of establishing and persisting in urban environments, despite high proportions of introduced species and poor‐quality soil conditions, which are indicative of many urban vacant sites (Bonthoux et al., 2011). Thus, to increase the relative proportion of native species in urban environments, management strategies should focus on enhancing native species recruitment into open habitats, via active planting and seeding efforts.

In contrast to local filtering effects, taxonomic and functional composition did change across study seasons, which supports our hypothesis (H3). Both metrics displayed strong compositional shifts in early seasons but exhibited different patterns between later seasons. Specifically, taxonomic composition showed no appreciable change in later seasons; although, functional composition continued to shift over time, indicating that native communities in our study exhibited similar trait responses regardless of seeded “community type,” local edaphic conditions, or potential competitive effects from spontaneous urban species. A long‐term experimental grassland study by Fukami et al. (2005) also found strong community convergence in functional composition over time, despite differences in the initial composition of species across treatments, which remained divergent over the nine‐year study period.

In contrast to local filtering effects, taxonomic and functional diversity indices differed across seasons, showing consistently lower diversity in season 3 than other sampled seasons, thereby also supporting our hypothesis (H3). By definition, successional dynamics are driven by the establishment of different plant species, with distinct (and often predictable) suites of functional strategies, over time. For example, succession in urban environments is characterized by shifts from annual and forb‐dominated communities in the first year, postdisturbance, to longer‐lived biennial and perennial, grass, and forb species in years two and three (Bazzaz, 1996). Thus, successional changes related to the establishment of functionally distinct communities likely explain the observed seasonal patterns in taxonomic and functional diversity in our study. CWM comparisons supported this conclusion, displaying an inverse relationship in the proportion of short‐lived annual species (decreasing) and longer‐lived biennials‐perennials (increasing) over time.

Seasonal differences in functional diversity (FDis) were driven by several traits. SLA, the proportion of C4 grasses, forbs, legumes, and annual and biennial‐perennial species all exhibited CWM differences across seasons. Seasonal trends tended to display either increasing (i.e., H_max_, legumes, and biennials‐perennials) or decreasing (i.e., Chl *a*, C4 grasses, annuals) CWM patterns over time. By contrast, trends in CWM SLA were cyclical, with SLA increasing during peak growth in June and decreasing in November during peak‐biomass. These results support the general conclusion that SLA is a highly variable trait that responds to a number of environmental factors, including the availability of light and water (Weiher et al., 1999; Westoby et al., 2002), which change predictably with season.

Community‐weighted mean seed weight was the only trait that did respond to soil environment or competition. Prior studies have shown that seed characteristics can rapidly change in response to strong urban selection pressures (Cheptou et al., 2008; Riba et al., 2005). These studies, however, were conducted on heterocarpic species (i.e., plants that produce two or more functionally different seed types), which only makeup a small pool of species commonly belonging to the Asteraceae family (Gardocki et al., 2000; Venable, 1985). Compared with leaf and whole‐plant functional characteristics, seed traits do not typically exhibit high degrees of trait variation (genetic and/or phenotypic plasticity; Violle et al., 2009). Additionally, seed weight is more likely to be influenced by dispersal‐related factors, such as recruitment limitation (McEuen & Curran, 2004) and high risk of dispersal (Cheptou et al., 2008)—factors which, logistically, could not be incorporated into our study design.

### 4.3 Changes in soil properties over time

Although native species in our study did not exhibit strong responses to soil environmental filtering for most biodiversity metrics (except Shannon diversity), soil analyses from seasons 1 and 4 showed that soil treatment groups (fill vs. topsoil) were characterized by different properties, which changed over time. Soil properties were highly variable among samples in season 1 with little differentiation between fill and topsoil treatment groups. By season 4, however, soil properties were more constrained among samples within each soil group, and there was strong divergence between soil treatment groups and seasons. Specifically, in season 1, both fill and topsoil were positively correlated with the majority of measured soil variables; however, fill samples tended to have higher concentrations of heavy metals, while soil moisture, organic matter, Pb, and soil macronutrient concentrations were more strongly associated with topsoil. Because both soil types used in our study were locally sourced, and possibly from the same locations in Maryland, it is not surprising that initial soil characteristics were similar between these treatment groups; although, physical properties, such as bulk density and texture, which were not measured as part of our soil analysis, would likely have differed (Scharenbroch et al., 2005). Over time, soil characteristics diverged between fill and topsoil samples. Myriad soil and trait‐related factors determine the availability and solubility of nutrients and contaminants for plant uptake (Cataldo & Wildung, 1978). Thus, a combination of physical and biological factors, including characteristics that influence soil water holding capacity and leaching, as well differences in nutrient and contaminant uptake rates by different species (Read et al., 2008), are likely responsible for the observed changes in soil conditions in our study over time. Although we identified distinct patterns in soil characteristics across seasons, it should be noted that observed patterns in our experimental study might not correspond to in situ comparisons of soil in urban environments, which are highly variable due to different contemporary management regimes and legacies of human influence (Johnson et al., 2015; Pouyat et al., 2010; Pouyat & Effland, 1999).

Abiotic and biotic soil characteristics can strongly influence both the survival of different plant species and the expression of functional traits in a community (Bernard‐Verdier et al., 2012). CWM comparisons showed that observed differences between soil treatment groups only influenced height (CWM H_max_), and the proportion of forbs and C4 grass species in our native communities. Interestingly, CWMs of these traits were greater in fill soil, indicating that native communities grew taller in fill soil plots and had higher proportions of forbs and C4 grass species. Assessing the effects of individual soil variables on functional trait values was not the intention of our study; thus, it is not clear which soil parameters had the greatest influence on individual trait values. However, lower soil moisture in fill soil plots likely had a strong filtering effect on our native communities, selecting species that are adapted to hotter, drier environments (i.e., species utilizing a C4 photosynthetic pathway; Barnes & Harrison, 1982).

Although native communities overall grew taller in fill soil plots (higher H_max_), the significant interaction between soil and season indicated that the effect of soil on H_max_ changed over time; that is, H_max_ of native species was higher in fill soil plots in early seasons (seasons 1 and 2), but shifted to topsoil in later seasons (seasons 3 and 4). This may be due to higher turnover rates of organic matter and soil nutrients in coarse‐textured soil (Van Veen et al., 1985; i.e., subsoil fill material), which increases the short‐term availability of macronutrients in fill soil. (Note‐soil texture was based on visual assessments only).

## 5 CONCLUSION

Numerous studies have focused on characterizing urban plant diversity patterns; however, this is the first study, to our knowledge, to experimentally assess the role of local assembly processes on native plant communities, in an effort to better understand the factors influencing both the structure and functioning of communities in urban habitats. We found that despite strong filtering effects of soil and competition from spontaneous urban species, native communities are able to successfully establish in experimental vacant habitats, as well as respond to environmental pressures via functional trait changes over time. These results suggest that regional factors related to dispersal limitation are responsible for the underrepresentation of native species in urban environments. Future research documenting and comparing seed dispersal pathways of both native and spontaneous urban species across a matrix of urban habitat patches will provide more evidence for identifying the relative influence of different assembly processes on urban plant community patterns. The strong seasonal effects found within our two‐year study period also suggest that urban communities undergo rapid changes over time. Therefore, research focused on a single time frame or season, likely does not capture the full scope of taxonomic and functional changes in a community. More long‐term studies of urban environments will not only provide a greater comprehensive view of urban communities, but it will also offer insights into the eco‐evolutionary dynamics of urban ecosystems over time.

## AUTHOR CONTRIBUTIONS

**Dorothy Borowy:** Conceptualization (lead); formal analysis (lead); methodology (lead); writing – original draft (lead). **Christopher M. Swan:** Conceptualization (supporting); project administration (equal); supervision (equal); writing – review and editing (equal).

## FUNDING INFORMATION

This research was jointly funded by the NSF Long‐term Ecological Research (LTER) Program (Grant No. DEB‐1027188) and the NSF Sustainability Research Network (SRN) Cooperative Agreement (Grant No DEB‐1444758). Any opinions, findings and conclusions or recommendations expressed in this material are those of the authors and do not necessarily reflect the views of the National Science Foundation.

## CONFLICT OF INTEREST

The authors declare that the research was conducted in the absence of any commercial or financial relationships that could be construed as a potential conflict of interest.

## Supporting information


Appendix S1

## Data Availability

The data presented in this study have been uploaded to Dryad (https://datadryad.org/stash/share/W_1PKTvtR2j76AwXzQ9‐Yh8MY3R4k6vuc5i672gO4j4).
