# Supplementary material for: The effects of local filtering processes on the structure and functioning of native plant communities in experimental urban habitats
Source: Ecol Evol. 2022 Oct 17;12(10):e9397. doi: 10.1002/ece3.9397 (PMC9575998; doi:10.1002/ece3.9397)
Supplement: Supplementary file 1 — Appendix S1 [file ECE3-12-e9397-s001.docx]

**APPENDIX. Supporting information: The effects of local filtering processes on the structure and functioning of native plant communities in experimental urban habitat**s

**Figure S1**. Photos of study plots taken in (A) June 2017 and (B) June 2018. Photo credit: D. Borowy

**(A)**

**(B)**

**Table S1**. List of seeded native species pool and associated functional groups and community type. Status column identifies species that successfully germinated and established in the experimental plots (✓); species that germinated but persisted in low densities (i.e., less than 5 individuals across all plots) (**-**); and species that did not germinate over the course of the study (X).

| Species | Growth Form | Photosynthetic Pathway | Life History | Primary Dispersal Mode | Community Type | Status |
| --- | --- | --- | --- | --- | --- | --- |
| *Andropogon virginicus* L. | Graminoid | C4 | Perennial | Wind | 2, 3, 4 | ✓ |
| *Asclepias tuberosa* L. | Forb | C3 | Perennial | Wind | 1, 6 | ✓ |
| *Aster laevis* L. | Forb | C3 | Perennial | Wind | 2, 5 | ✓ |
| *Bidens frondosa* L. | Forb | C3 | Annual | Attachment | 1, 4 | ✓ |
| *Chamaecrista fasciculata* (Michx.) Greene | Forb-legume | C3 | Annual | Ballistic | 3, 6 | ✓ |
| *Coreopsis* *verticillata* Ehrh. | Forb | C3 | Perennial | Unassisted/bird | 1, 4, 6 | ✓ |
| *Desmodium paniculatum* (L.) DC. | Forb-Legume | C3 | Perennial | Attachment | 2, 3, 5 | ✓ |
| *Elymus histrix* L. | Graminoid | C3 | Perennial | Attachment | 1, 2 | ✓ |
| *Eupatorium altissimum* L. | Forb | C3 | Perennial | Wind | 3, 6 | ✓ |
| *Heliopsis helianthoides* (L.) Sweet | Forb | C3 | Perennial | Bird | 2, 5 | ✓ |
| *Lespedeza capitata* Michx. | Forb-Legume | C3 | Perennial | Bird | 1, 6 | ✓ |
| *Liatris spicata* (L.) Willd. | Forb | C3 | Perennial | Wind | 2, 4 | ✓ |
| *Monarda punctata* L. | Forb | C3 | Annual | Unassisted | 3, 4, 6 | ✓ |
| *Oenothera fruticosa* L. | Forb | C3 | Biennial-Perennial | Wind | 1, 4 | X |
| *Oxalis violacea* L. | Forb | C3 | Perennial | Unassisted-ballistic | 2, 5 | ➖ |
| *Penstemon hirsutus* (L.) Willd. | Forb | C3 | Perennial | Unassisted | 2, 6 | ✓ |
| *Rudbeckia hirta* L. | Forb | C3 | Annual | Bird | 2, 4, 5 | ✓ |
| *Schizachyrium scoparium* (Michx.) Nash | Graminoid | C4 | Perennial | Wind | 1, 4, 5 | ✓ |
| *Silene stellata* (L.) W.T. Aiton | Forb | C3 | Perennial | Unassisted/wind | 1, 5 | X |
| *Solidago odora* Aiton | Forb | C3 | Perennial | Wind | 3, 5 | X |
| *Sorghastrum nutans* (L.) Nash | Graminoid | C4 | Perennial | Wind | 3, 5, 6 | ✓ |
| *Thalictrum thalictroides* (L.) A.J. Eames & B. Boivin | Forb | C3 | Perennial | Ant | 3, 4, 5, 6 | ➖ |
| *Tridens flavus* (L.) Hitchc. | Graminoid | C3 | Perennial | Attachment | 3, 6 | ✓ |
| *Trillium grandiflorum* (Michx.) Salisb. | Forb | C3 | Perennial | Ant | 1, 3 | X |
| *Viola sagittata* Aiton | Forb | C3 | Perennial | Ant | 1, 2, 4 | ✓ |

**Table S2**. Mean and standard errors of (A) season 1 and (B) season 4 soil variables.

| (A) | Fill | | | Topsoil | | |
| --- | --- | --- | --- | --- | --- | --- |
| Soil Variable | Mean ± SE | Min | Max | Mean ± SE | Min | Max |
| Moisture (%) | 0.86 ± 0.04 | 0.72 | 1.14 | 1.04 ± 0.03 | 0.78 | 1.20 |
| pH | 7.68 ± 0.04 | 7.37 | 7.86 | 7.62 ± 0.04 | 7.32 | 7.80 |
| Organic matter (%) | 2.94 ± 0.04 | 2.72 | 3.14 | 3.09 ± 0.05 | 2.84 | 3.38 |
| Aluminum (mg/K) | 26.54 ± 0.84 | 23.97 | 32.54 | 24.6 ± 0.30 | 23.46 | 26.91 |
| Arsenic (mg/K) | 0.14 ± 0.01 | 0.07 | 0.18 | 0.18 ± 0.002 | 0.18 | 0.20 |
| Calcium (mg/K) | 2573 ± 59.79 | 2226 | 3011 | 2720 ± 127.99 | 2376 | 4002 |
| Cadmium (mg/K) | 0.33 ± 0.03 | 0.19 | 0.49 | 0.29 ±  0.03 | 0.21 | 0.60 |
| Chromium (mg/K) | 0.13 ± 0.01 | 0.08 | 0.16 | 0.08 ± 0.01 | 0.06 | 0.13 |
| Copper (mg/K) | 0.42 ± 0.02 | 0.26 | 0.54 | 0.33 ± 0.03 | 0.23 | 0.70 |
| Iron (mg/K) | 19.29 ± 2.35 | 5.84 | 32.49 | 6.62 ± 1.09 | 4.33 | 14.81 |
| Potassium (mg/K) | 104.35 ± 2.26 | 90.7 | 116.4 | 105.9 ± 1.66 | 92.58 | 114.17 |
| Magnesium (mg/K) | 266.96 ± 18.08 | 214.1 | 417.4 | 319.07 ± 11.02 | 232.20 | 354.10 |
| Manganese (mg/K) | 14.84 ± 0.62 | 11.88 | 18.04 | 12.98 ± 0.29 | 11.32 | 14.21 |
| Sodium (mg/K) | 53.00 ± 1.49 | 46.49 | 66.43 | 48.66 ± 1.07 | 42.80 | 56.24 |
| Phosphorus (mg/K) | 2.67 ± 0.1 | 2.15 | 3.23 | 2.61 ± 0.06 | 2.08 | 2.88 |
| Lead (mg/K) | 1.12 ± 0.05 | 0.93 | 1.49 | 1.49 ± 0.07 | 0.80 | 1.83 |
| Zinc (mg/K) | 1.18 ± 0.08 | 0.65 | 1.56 | 0.99 ± 0.11 | 0.71 | 1.95 |

| (B) | Fill | | | Topsoil | | |
| --- | --- | --- | --- | --- | --- | --- |
| Soil Variable | Mean ± SE | Min | Max | Mean ± SE | Min | Max |
| Moisture (%) | 0.64 ± 0.03 | 0.49 | 0.86 | 1.04 ± 0.03 | 0.66 | 0.87 |
| pH | 7.54 ± 0.08 | 6.91 | 7.79 | 7.62 ± 0.04 | 7.16 | 7.80 |
| Organic matter (%) | 3.02 ± 0.12 | 2.47 | 4.03 | 3.09 ± 0.05 | 2.69 | 3.42 |
| Aluminum (mg/K) | 16.70 ± 0.70 | 14.86 | 23.22 | 24.65 ± 0.30 | 12.80 | 17.01 |
| Arsenic (mg/K) | 0.15 ± 0.007 | 0.09 | 0.17 | 0.19 ± 0.002 | 0.17 | 0.21 |
| Calcium (mg/K) | 2061 ± 76.35 | 1591 | 2592 | 2720 ± 127.99 | 1632 | 2555 |
| Cadmium (mg/K) | 0.32 ± 0.02 | 0.21 | 0.42 | 0.29 ± 0.03 | 0.18 | 0.25 |
| Chromium (mg/K) | 0.10 ± 0.005 | 0.08 | 0.14 | 0.08 ± 0.006 | 0.05 | 0.07 |
| Copper (mg/K) | 0.31 ± 0.019 | 0.20 | 0.44 | 0.33 ± 0.04 | 0.15 | 0.25 |
| Iron (mg/K) | 8.55 ± 0.52 | 4.74 | 11.30 | 6.62 ± 1.09 | 2.60 | 3.41 |
| Potassium (mg/K) | 99.35 ± 7.6 | 70.40 | 159.92 | 105.91 ± 1.66 | 68.91 | 145.99 |
| Magnesium (mg/K) | 201.82 ± 3.51 | 176.80 | 220.60 | 319.08 ± 11.01 | 192.30 | 293.80 |
| Manganese (mg/K) | 9.44 ± 0.64 | 6.69 | 15.08 | 12.98 ± 0.29 | 6.04 | 10.70 |
| Sodium (mg/K) | 29.27 ± 0.50 | 26.61 | 31.76 | 48.66 ± 1.07 | 28.79 | 32.06 |
| Phosphorus (mg/K) | 3.05 ± 0.14 | 2.32 | 3.91 | 2.61 ± 0.06 | 2.22 | 3.96 |
| Lead (mg/K) | 0.78 ±  0.04 | 0.58 | 1.06 | 1.49 ± 0.07 | 1.06 | 1.36 |
| Zinc (mg/K) | 0.70 ± 0.04 | 0.48 | 0.97 | 0.99 ± 0.11 | 0.33 | 1.51 |

**Table S3**. Pearson’s correlation coefficients and associated p-values of soil variables for each PCA axis.

|  | PCA Axis | Pearson’s r | P |
| --- | --- | --- | --- |
| Aluminum (Al) | 1 | 0.87 | 4.51E-16 |
| Arsenic (As) | 1 | -0.48 | 5.51E-04 |
| Cadmium (Cd) | 1 | 0.46 | 1.04E-03 |
| Calcium (Ca) | 1 | 0.46 | 9.31E-04 |
| Chromium (Cr) | 1 | 0.76 | 5.35E-10 |
| Copper (Cu) | 1 | 0.78 | 6.31E-11 |
| Iron (Fe) | 1 | 0.82 | 1.05E-12 |
| Manganese (Mn) | 1 | 0.90 | 6.26E-18 |
| Moisture | 1 | 0.32 | 2.61E-02 |
| pH | 1 | 0.38 | 7.42E-03 |
| Sodium (Na) | 1 | 0.85 | 2.48E-14 |
| Zinc (Zn) | 1 | 0.73 | 3.22E-09 |
| Arsenic (As) | 2 | 0.51 | 1.88E-04 |
| Cadmium (Cd) | 2 | -0.47 | 7.23E-04 |
| Calcium (Ca) | 2 | 0.67 | 1.93E-07 |
| Chromium (Cr) | 2 | -0.53 | 9.39E-05 |
| Iron (Fe) | 2 | -0.44 | 2.00E-03 |
| Lead (Pb) | 2 | 0.82 | 9.48E-13 |
| Magnesium (Mg) | 2 | 0.96 | 5.02E-27 |
| Moisture | 2 | 0.89 | 3.76E-17 |
| Organic matter (OM) | 2 | 0.42 | 3.26E-03 |
| Potassium (K) | 2 | 0.40 | 4.71E-03 |
| Sodium (Na) | 2 | 0.42 | 2.85E-03 |
|  |  |  |  |
